# Intestinal permeability of agaro-oligosaccharides: Transport across Caco-2 cell monolayers and pharmacokinetics in rats

**DOI:** 10.3389/fnut.2022.996607

**Published:** 2022-09-16

**Authors:** Ikuya Shirai, Koji Karasawa, Yusuke Kodaira, Yu Iwasaki, Yasutaka Shigemura, Hidefumi Makabe, Shigeru Katayama

**Affiliations:** ^1^Department of Science and Technology, Graduate School of Medicine, Science and Technology, Shinshu University, Nagano, Japan; ^2^Ina Food Industry Co., Ltd., Ina, Japan; ^3^Department of Biomolecular Innovation, Institute for Biomedical Sciences, Shinshu University, Nagano, Japan; ^4^Faculty of Domestic Science, Tokyo Kasei University, Tokyo, Japan

**Keywords:** agaro-oligosaccharide, Caco-2, intestinal permeability, paracellular pathway, agarobiose

## Abstract

Agaro-oligosaccharides (AOSs), even-numbered oligosaccharides prepared from agar, are applied to various food, including supplements, drinks, and jellies because of their biological activities. This study aimed to evaluate the AOS permeation in the gastrointestinal tract *in vivo* and *in vitro*. Agarobiose (Abi), agarotetraose (Ate), and agarohexaose (Ahe) were detected in rat plasma after oral administration of AOSs. The detection level of agarobiose in the plasma was higher than that of agarohexaose, which was consistent with the permeation study using Caco-2 cell monolayers. Further, the adenosine triphosphate inhibitor (sodium azide) or endocytosis inhibitor (colchicine) did not inhibit AOS permeation through Caco-2 cell monolayers. Conversely, AOS permeation enhanced upon treatment with cytochalasin B, a tight junction disrupter, suggesting that AOSs might have passed mainly through the tight junctions between the intestinal epithelial cells. These results indicate that AOSs, especially agarobiose, can be absorbed as an intact form *via* the gastrointestinal tract across the intestinal epithelium through the paracellular pathway.

## Introduction

Agar, a linear galactan extracted from red seaweed, has been consumed in Asian countries for a long time. Agarose, a major polysaccharide in agar, consists of alternating (1 → 3)-linked β-d-galactose (Gal) and (1 → 4)-linked 3,6-anhydro-α-l-galactose (AnGal) residues (1). Two kinds of oligosaccharides can be produced from agar depending on the type of sugar at the reducing end: agaro-oligosaccharides (AOSs) or neoagaro-oligosaccharides. Under mildly acidic conditions, the α-(1 → 3)-galactosidic bond of agarose is hydrolyzed to AOSs composed of repeating agarobiose (Abi) units with AnGal at the reducing end (2).

Agaro-oligosaccharides have attracted considerable attention in the food, cosmetic, and pharmaceutical industries because of their biological effects, including antioxidant (3, 4), anti-inflammatory (5), anti-tumor (6), and anti-obesity (7) activities. Regarding their effects on intestinal function, several studies have demonstrated that orally administered AOSs attenuate high-fat-diet-induced gut dysbiosis (8) and prevent non-steroidal anti-inflammatory drug-induced small intestinal injury in mice (9) while other studies have shown its biological effects on the peripheral tissues. Enoki et al. (6) reported that orally administered AOSs decreased the edemas of the 12-*O*-tetradecanoylphorbol-13-acetate-induced ear edema model mice, suggesting that AOSs could translocate from the gastrointestinal tract into the circulating blood and directly reduce inflammatory reactions in peripheral tissues. However, it still remains unclear whether orally administered AOSs permeate across the intestinal epithelium and are absorbed as an intact form.

Caco-2 cells are derived from human colon adenocarcinoma cell line, but this cell line can spontaneously differentiate to express morphological and functional characteristics of mature small intestinal enterocytes, such as microvilli, tight junctions, and some important transporters on the apical membrane (10). Therefore, Caco-2 cells are widely used for *in vitro* studies to investigate the intestinal permeability and their transport mechanisms of drugs and food compounds.

In this study, we aimed to measure the plasma concentrations of AOSs after its oral administration in rats. The blood plasma of the rats orally administered with AOSs was collected and labeled with *p*-aminobenzoic ethyl ester (ABEE). The concentrations of Abi, agarotetraose (Ate), and agarohexaose (Ahe) were measured by liquid chromatography mass spectrometry (LC-MS). In addition, an *in vitro* permeation study was performed to identify the intestinal transport mechanism of the AOSs across the Caco-2 cell monolayers using different transport inhibitors.

## Materials and methods

### Chemicals and reagents

Eagle's minimal essential medium (E-MEM), MEM non-essential amino acids (MEM-NEAA) solution, penicillin–streptomycin solution (PS), Hank's balanced salt solution (HBSS), 0.25% w/v trypsin-1 mmol/L ethylenediamine tetraacetic acid·4 Na solution, ethanol, dimethyl sulfoxide (DMSO), sodium chloride, potassium chloride, disodium hydrogen phosphate, potassium dihydrogen phosphate, chloroform, 3-(4,5-dimethylthiazol-2-yl)-2,5-diphenyltetrazolium bromide (MTT), sodium azide, colchicine, cytochalasin B, heparin sodium, and Lucifer yellow CH dilithium salt fluorescent stain (LY) were purchased from FUJIFILM Wako Pure Chemical Corporation (Osaka, Japan). Fetal bovine serum (FBS) was purchased from Merck (Darmstadt, Germany). Acetonitrile was purchased from Nacalai Tesque Inc. (Tokyo, Japan).

### Isolation of Abi, Ate, and Ahe

The AOSs were prepared by the acid hydrolysis of agar according to the method described by Enoki et al. (5) with slight modification. In brief, agar was suspended in 0.1 N HCl to 10% (w/v) and heated at 90°C for 30 min. After being cooled to room temperature, powdered activated carbon was added and incubated for 60 min. Powdered activated carbon was filtered and the AOSs solution was freeze dried. The oligosaccharides of different sizes were isolated from the AOSs through size exclusion chromatography using a flash column (15 mm × 170 cm) packed with the Toyopearl HW-40S (Tosoh, Tokyo, Japan) stationary phase at a mobile phase [distilled water (DW)] flow rate of 0.1 ml/min to fractionate the AOSs with different molecular weights (MWs). The obtained each fraction was analyzed with HPLC-refractive index detector (RID-10A, Shimazu, Kyoto, Japan) system. Each fraction was separated on a TSKgel G2500 PWXL (300 × 7.8 mm, 7.0 μm; Showa Denko K.K., Tokyo, Japan). The mobile phase was DW and sent using LC-10AT pump (Shimazu). The fractions were collected and freeze-dried, and then their chemical structures were determined by nuclear magnetic resonance (NMR) spectroscopy to identify Abi, Ate, and Ahe. ^1^H and ^13^C NMR spectra (Supplementary Figures 1, 2) were recorded on a Bruker Avance 500 MHz spectrometer in D_2_O, and chemical shifts of the ^13^C NMR spectra of Abi and Ate were assigned (Supplementary Table 1).

### ABEE derivatization of AOSs

To prepare the standard curve samples of AOSs, the AOSs were labeled with ABEE using an ABEE labeling kit (J-Chemical, Tokyo, Japan) according to the method developed by Yasuno et al. (11). The reaction was performed according to the instructions provided by the manufacturer. Briefly, 40 μl of the ABEE reagent solution was added to 10 μl of the solution of AOSs and the mixture was vortexed and incubated at 80°C for 1 h. After cooling to 15–20°C, 200 μl of DW and an equal volume of chloroform were added, and the mixture was then vortexed and centrifuged for 1 min. The upper layer was filtered through a polytetrafluoroethylene membrane (4 mm diameter, 0.45-μm pore size; Membrane Solutions Limited, Plano, TX, USA) and subjected to LC-MS.

### Quantification of ABEE-derivatized AOSs by LC-MS

For the HPLC measurements, an LC-20AD pump (Shimadzu) fitted with a DGU-20A 5R degassing unit (Shimadzu) was used. The analytes were injected using a SIL-20AC autosampler (Shimadzu). In addition, a CTO-20AC column oven (Shimadzu) was used, and entire equipment was controlled using a CBM-20A unit (Shimadzu). The samples were separated on a Shim-pack VP-ODS (150 × 2.0 mm, 4.6 μm; Shimadzu) combined with a Shim-pack GVP-ODS guard column (2.0 mm i.d. × 5 mm, 4.6 μm; Shimadzu). The mobile phase consisted of acetic acid (0.1%) in water (A) and acetic acid (0.1%) in acetonitrile (B). The gradient conditions were as follows: 0–30 min, 9%−13% B; 30–45 min, 80% B; 45–60 min, 9% B. The flow rate was 0.2 ml/min. The column oven temperature was set at 60°C, and the autosampler temperature was 10°C.

The detection was performed using an LCMS-2020 (Shimadzu) ESI-MS device. The analytes were identified and quantified by selected ion monitoring in the positive-ion mode. The MS conditions were as follows: interface temperature, 350°C; desolvation line (DL) temperature, 250°C; heat block temperature, 200°C; nebulizer gas flow, 1.5 L/min; drying gas flow, 15 L/min; interface voltage, 4.5 kV; DL voltage, 0 kV.

### Animals

Fifteen female Sprague–Dawley rats, aged 7 weeks, were purchased from Charles River Laboratories Japan (Kanagawa, Japan). The rats were acclimatized to standard laboratory conditions (20–25°C, 40%−70% humidity, and 12 h light/dark cycle) for 1 week. All animals had free access to tap water and standard rodent chow (MF; Oriental Yeast Co., Ltd., Tokyo, Japan). All experimental protocols were approved by the Institutional Animal Care and Use Committee of Shinshu University (Permission No. 020062).

### *In vivo* absorption tests

After a fasting period of 12 h, 2 ml of phosphate-buffered saline (pH 7.4) containing 0%, 10%, or 50% AOSs was orally administered to five rats from each group (220–250 g body weight) using feeding gavages. The AOSs comprised 30.2, 29.1, and 31.6% of Abi, Ate, and Ahe, respectively. Blood (100 μl) was collected before and after 1, 2, 4, 8, and 24 h of oral administration from the tail vein using 10,000 U heparin-coated syringes (1 ml) with a 26G × $\frac{1}{2}$” needle. The blood samples were centrifuged at 1,000 g for 10 min, and the supernatants were collected. Acetonitrile was added to the plasma samples at a final concentration of 60%. The samples were vigorously vortexed and centrifuged at 6,000 g for 5 min; the supernatants were collected and evaporated to dryness. Subsequently, the dried samples were dissolved in 10 μl of DW and subjected to ABEE derivatization before LC-MS analysis. The area under the curve (AUC) of plasma concentration was calculated using the trapezoid formula.

### Cell culture for permeation experiments

Caco-2 cells were purchased from DS Pharma (Osaka, Japan). The cells (passage 20–27) were cultured in E-MEM containing 10% (v/v) FBS, 1% (v/v) MEM-NEAA, and 1% (v/v) PS. The cells were incubated at 37°C in an atmosphere of 5% CO_2_ under 95% relative humidity. When the cells reached 80–90% confluence, they were passaged at a density of 1.0 × 10^5^ cells onto a 12-well Transwell Permeable Support (0.4-μm pore polyester membrane insert, Corning, NY, USA). The cells were cultured for 19–21 days to obtain a monolayer. The culture medium (0.6 ml in the apical side and 1.5 ml in the basolateral side) was replaced every 1–2 days. The integrity of the Caco-2 cell monolayer was evaluated by measuring transepithelial electrical resistance (TEER) using a Millicell-ERS-2 system (Merck, Darmstadt, Germany). Only cell monolayers with final TEER values above 700 Ω · cm^2^, indicative of high barrier integrity, were used for the transport assays.

### MTT assay

The cell viability and survivability were determined using an MTT assay. Caco-2 cells were cultured in 96-well microtiter plates at 1 × 10^5^ cells/well in 0.2 ml E-MEM at 37°C. When the cells reached 70% confluence, the culture medium was removed and replaced with different concentrations of Abi or Ahe (0.125, 0.25, 0.5, and 1 mM) in 100 μl of HBSS solution. The medium was removed after incubating for 6 h, and 0.15 ml of 0.5 mg/ml MTT solution in HBSS was added to each well. After incubating in the dark for 4 h, the medium was removed, and 0.15 ml of DMSO was added to each well to dissolve formazan crystals, with gentle shaking for 15 min at 37°C. The absorbance was measured at 570 nm using a Nivo Multimode Plate Reader (PerkinElmer, Waltham, MA, USA).

### Transepithelial permeation experiment

Caco-2 cells monolayers were preincubated in HBSS for 30 min at 37°C. Subsequently, 0.6 ml of 0.5 mg/ml Abi and Ahe and 1.5 ml of HBSS to the apical and basolateral sides, respectively. After incubation for 0, 1, 2, and 3 h, the supernatant was collected from the basolateral side. To identify the transport mechanisms, Caco-2 cell monolayers were preincubated with sodium azide (1 mM), colchicine (1 mM), or cytochalasin B (5 μg/ml) dissolved in HBSS with 0.1% DMSO for 30 min, followed by the incubation of 0.5 mg/ml Abi and Ahe for 2 h. To remove salt, the collected supernatant was applied to the graphitized carbon solid-phase extraction (GC-SPE) using InertSep GC (200 mg/3 ml) (GL Sciences Inc., Tokyo, Japan), according to the method described by Ninonuevo et al. (12). The SPE cartridge was first equilibrated with 6 ml of 80% acetonitrile in 0.1% acetic acid (v/v), followed by 3 ml of DW. The basal solution was dissolved in DW to 3 ml and loaded onto the cartridge. The salts were removed by washing with 6 ml of DW, and Abi or Ahe was eluted with 3 ml of 20% acetonitrile. After ABEE derivatization, the collected solution was evaporated to dryness and subjected to LC-MS. The apparent permeability coefficients (*Papp*) were calculated (13) as *Papp* = (d*Q*/d*t*) × (1/*A*C_0_), where d*Q*/d*t* is the permeation rate (μg/s), *A* is the area of the monolayer of Caco-2 cells (cm^2^), and *C*_0_ is the initial concentration of Abi or Ahe in the apical chamber (μg/ml).

### Statistics

Data are presented as the mean ± standard error. The data from the MTT assay were compared using the one-way analysis of variance (ANOVA). The data from the permeation experiment were also compared using one-way ANOVA or the Student's *t*-test. The differences were considered significant at *P* < 0.05. Statcel 4 (OMS Publishing Co., Saitama, Japan) was used for statistical analysis.

## Results

### Preparation of standard curve of ABEE-derivatized AOSs

**Figure 1:**
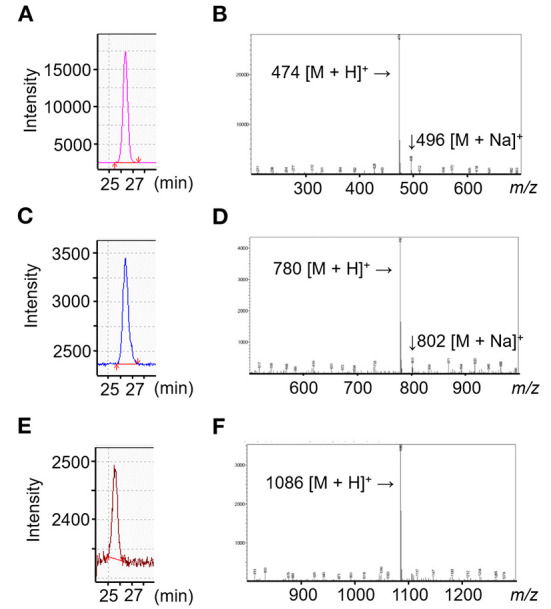
Liquid chromatography-mass spectrometry (LC-MS) results of agaro-oligosaccharides (AOSs). **(A–C)** MS chromatogram of *p*-aminobenzoic ethyl ester-derivatives of agarobiose, agarotetraose, and agarohexaose (ABEE-Abi, ABEE-Ate, ABEE-Ahe). In the selected ion monitoring mode, ABEE-Abi **(A)**, ABEE-Ate **(B)**, and ABEE-Ahe **(C)** were detected at *m/z* 474, 780, and 1,086, respectively. **(D–F)** MS spectra of ABEE-AOSs. Base peaks of ABEE-Abi **(D)**, ABEE-Ate **(E)**, and ABEE-Ahe **(F)** were protonated ions.

The ABEE labeling method was applied to achieve sufficiently high sensitivity for detecting AOSs both *in vivo* and *in vitro*. The ABEE-derivatives of Abi, Ate, and Ahe, denoted as ABEE-Abi, ABEE-Ate, and ABEE-Ahe (Supplementary Figure 3), respectively, were prepared as the standard curve samples. The mass chromatograms and mass spectra obtained for a 5 pmol standard solution of ABEE-Abi, ABEE-Ate, and ABEE-Ahe are illustrated in Figure 1. In the positive-ion mode, the base mass peaks of ABEE-Abi, ABEE-Ate, and ABEE-Ahe were observed at *m/z* 474.3, 780.3, and 1086.3, corresponding to the protonated molecules. The sodium adducts of ABEE-Abi and ABEE-Ate were also detected at *m/z* 496.3 and 802.3, respectively. The linearity of the standard curve ranging from 0.2 to 10 pmol was confirmed for ABEE-Abi, ABEE-Ate, and ABEE-Ahe (*R*^2^ ≥ 0.999) (Supplementary Figure 4).

### AOSs levels in the rat plasma after oral administration

**Figure 2:**
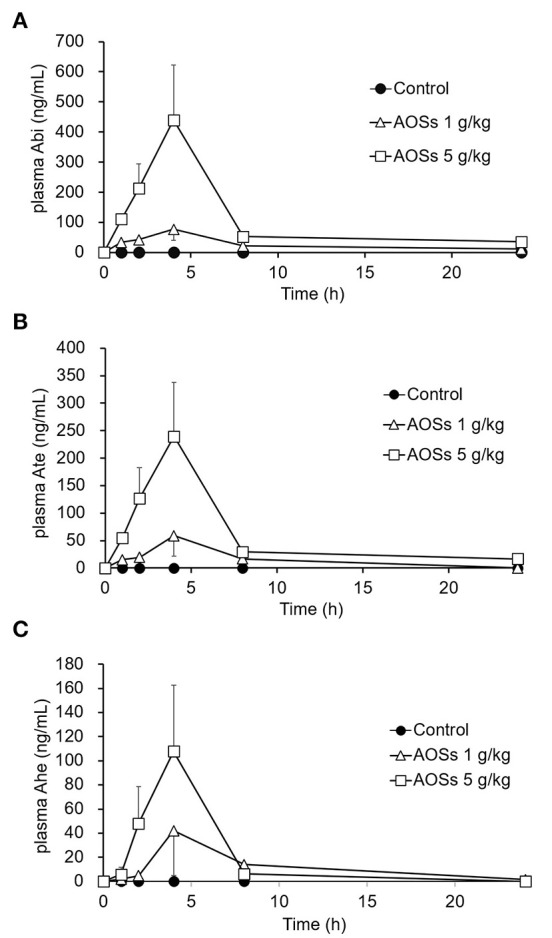
Plasma concentration–time profiles of Abi **(A)**, Ate **(B)**, and Ahe **(C)** after oral administration of AOSs to rats. Each value represents the mean ± standard error (SE) (*n* = 5).

The absorption of AOSs into the bloodstream of rats after oral administration was examined. After administering 1 or 5 g/kg AOSs, the concentrations of Abi, Ate, and Ahe in the rat plasma increased and reached a maximum value after 4 h (Figure 2). The levels of AOSs detected in the plasma of the rats administered with 5 g/kg AOSs were higher compared with those administered with 1 g/kg AOSs. The AUC and maximum observed plasma concentration (Cmax) values of Abi, Ate, and Ahe in the plasma of the rats administered with 5 g/kg AOSs were higher than those of 1 g/kg AOS (Table 1). The highest AUC and Cmax values were observed in Abi detection, and AOSs with higher MWs were detected to a lower level in the rat plasma. There were no significant differences in Tmax between AOSs with different MWs.

**Table 1:** Pharmacokinetic parameters of orally-administered AOSs in rat plasma.

**AOSs**	**1 g/kg**			**5 g/kg**		
	**Tmax**	**Cmax**	**AUC**	**Tmax**	**Cmax**	**AUC**
	**(h)**	**(ng/ml)**	**(μg·h/ml)**	**(h)**	**(ng/ml)**	**(μg·h/ml)**
Abi	4	76.4 ± 36.7	0.62 ± 0.18	4	439.8 ± 182.6	2.56 ± 0.81
Ate	4	58.9 ± 37.6	0.39 ± 0.17	4	239.5 ± 98.2	1.39 ± 0.45
Ahe	4	42.1 ± 37.1	0.29 ± 0.27	4	107.8 ± 55.0	0.46 ± 0.25

AOSs, agaro-oligosaccharides; Abi, agarobiose; Ate, agarotetraose; Ahe, agarohexaose; Tmax, time at maximal concentration; Cmax, maximum observed plasma concentration; AUC, area under the curve.

### Effects of Abi and Ahe on Caco-2 cell viability

**Figure 3:**
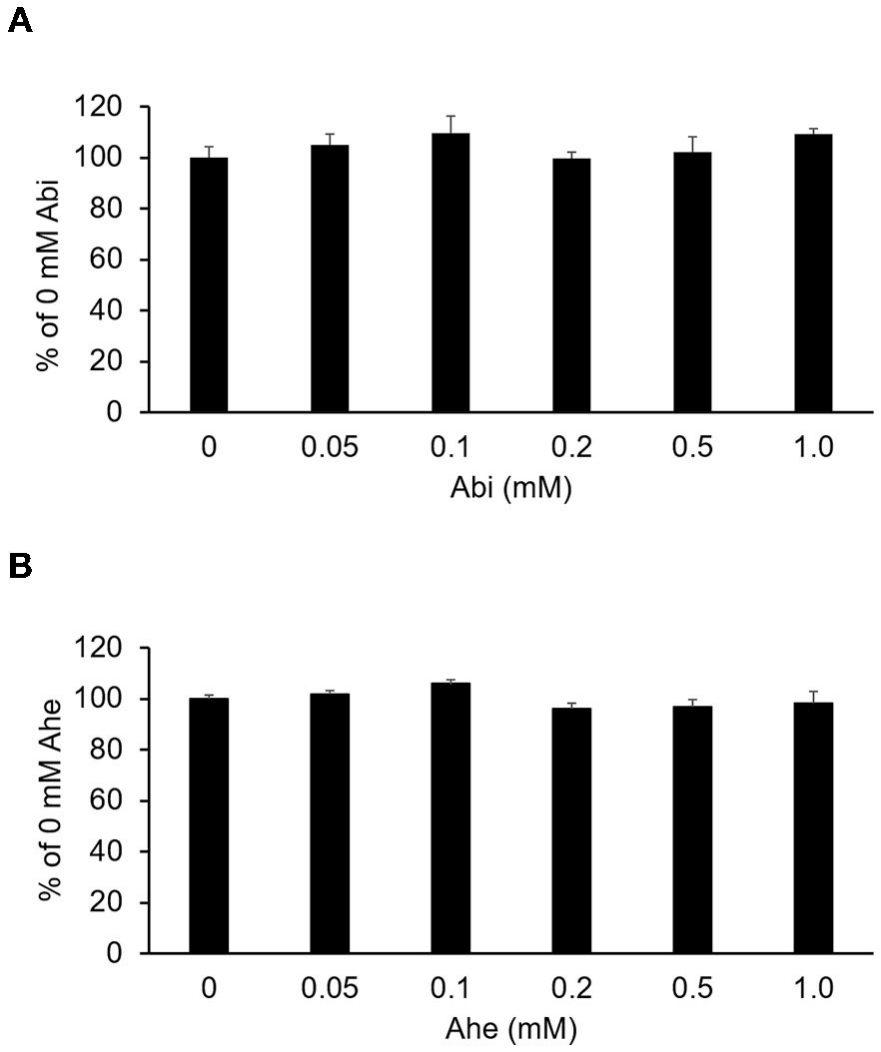
Cytotoxicity of Abi **(A)** and Ahe **(B)** on Caco-2 cells was determined using the 3-(4,5-dimethylthiazol-2-yl)-2,5-diphenyltetrazolium bromide assay after 6 h of incubating Caco-2 cells with Hank's balanced salt solution containing Abi or Ahe. Each value represents the mean ± SE (*n* = 5).

The MTT assay was conducted to evaluate the effects of Abi and Ahe on the Caco-2 cell viability. As shown in Figure 3, Abi and Ahe did not exhibit cytotoxic effects on Caco-2 cells at concentrations up to 1 mM. Therefore, 0.5 mM of Abi and Ahe was used in the subsequent experiments.

### Transepithelial permeation of Abi and Ahe

**Figure 4:**
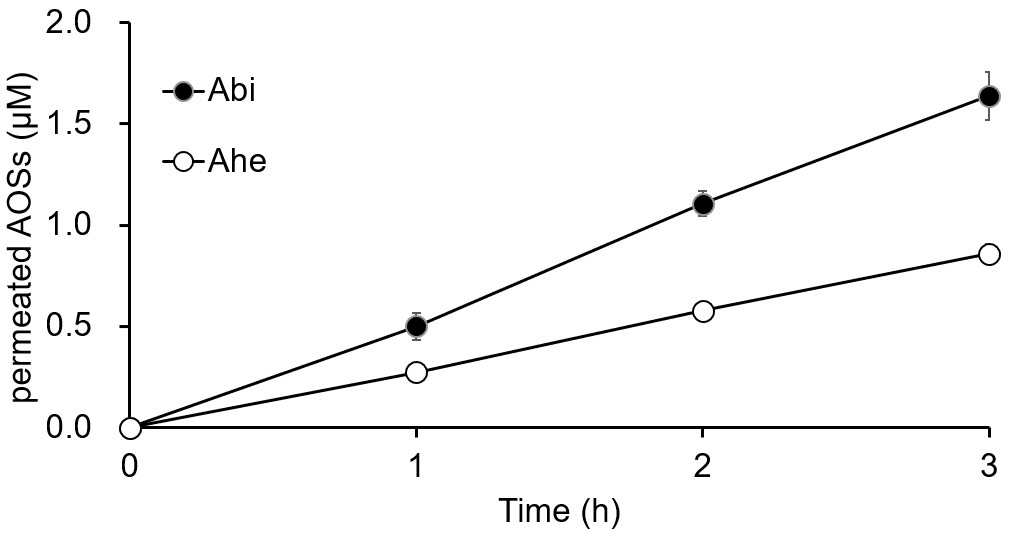
The cumulative amounts of Abi and Ahe that permeated the Caco-2 cell monolayers. Abi and Ahe were added to the apical chamber of the Transwell insert wherein the Caco-2 cell monolayers were cultured. Subsequently, the basolateral solution was taken, and the amount of permeated Abi or Ahe was quantified using LC-MS. Each value represents the mean ± SE (*n* = 3).

The Caco-2 cell monolayers cultured on Transwell inserts for 19–21 days were used for the *in vitro* permeation experiments. Both Abi and Ahe permeated the Caco-2 cell monolayers, and the quantity permeated increased linearly with the increasing incubation time (Figure 4). *Papp* (10^−6^ cm/s) of Abi and Ahe was 0.41 ± 0.03 and 0.21 ± 0.01, respectively; the percentage of Abi and Ahe permeated was 0.27 ± 0.02 and 0.14 ± 0.01%/h, respectively.

### Analysis of the transepithelial permeation route of Abi and Ahe

**Figure 5:**
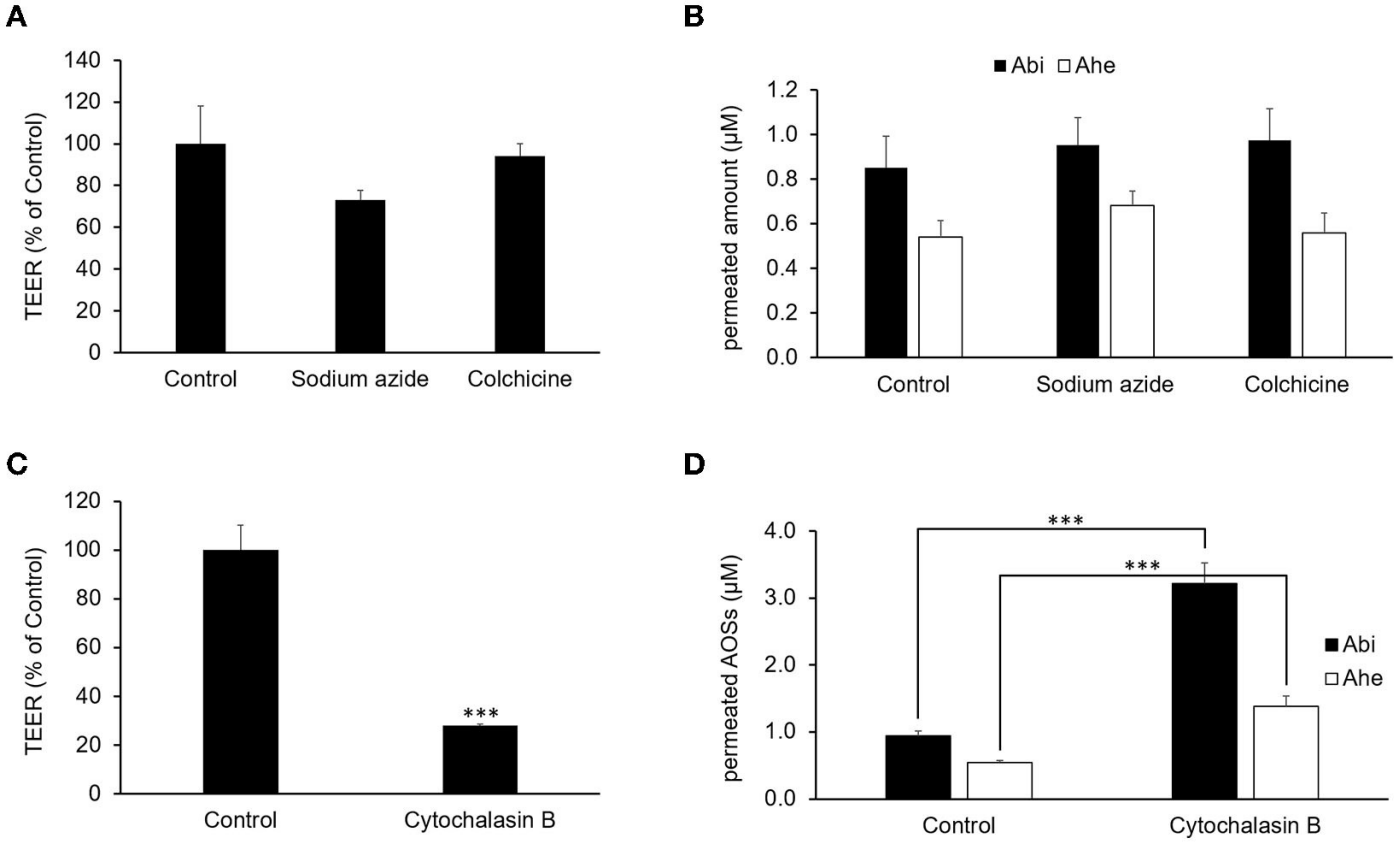
Effects of sodium azide, colchicine, and cytochalasin B on the permeability of AOSs across the Caco-2 cell monolayers. Sodium azide, colchicine, or cytochalasin B was added to the apical chamber 30 min before adding Abi and Ahe. After incubation for 2 h, the TEER **(A,C)** and amount of permeated Abi and Ahe in the basolateral chamber **(B,D)** were measured. Each value represents the mean ± SE (*n* = 3). The three asterisks (***) correspond to *P* < 0.001 vs. the control.

The effects of various inhibitors on the transepithelial absorption through Caco-2 cell monolayers were examined to identify the transport routes of Abi and Ahe. We used 1 mM sodium azide and 1 mM colchicine as an electron-transport-chain inhibitor and a transcytosis inhibitor, respectively, to determine whether an energy-dependent pathway is involved. The inhibitors did not have any significant effect on the TEER values or the transport rates of Abi and Ahe (Figures 5A,B). These results suggest that an energy-dependent route was not involved in the transepithelial permeation of AOSs. Next, we used cytochalasin B as a tight junction disruptor to investigate the involvement of the paracellular pathway in the transepithelial permeation of Abi and Ahe. Cytochalasin B increases the paracellular permeability by affecting the tight junction structure through the myosin light chain kinase activation (14). Treatment with 5 μg/ml cytochalasin B significantly decreased the TEER value of the Caco-2 cell monolayers and increased the transepithelial permeability of Abi and Ahe (Figures 5C,D), indicating that Abi and Ahe mainly permeate through the Caco-2 cell monolayers *via* the paracellular pathway.

### Correlation between TEER and transepithelial permeation rate of Abi and Ahe

**Figure 6:**
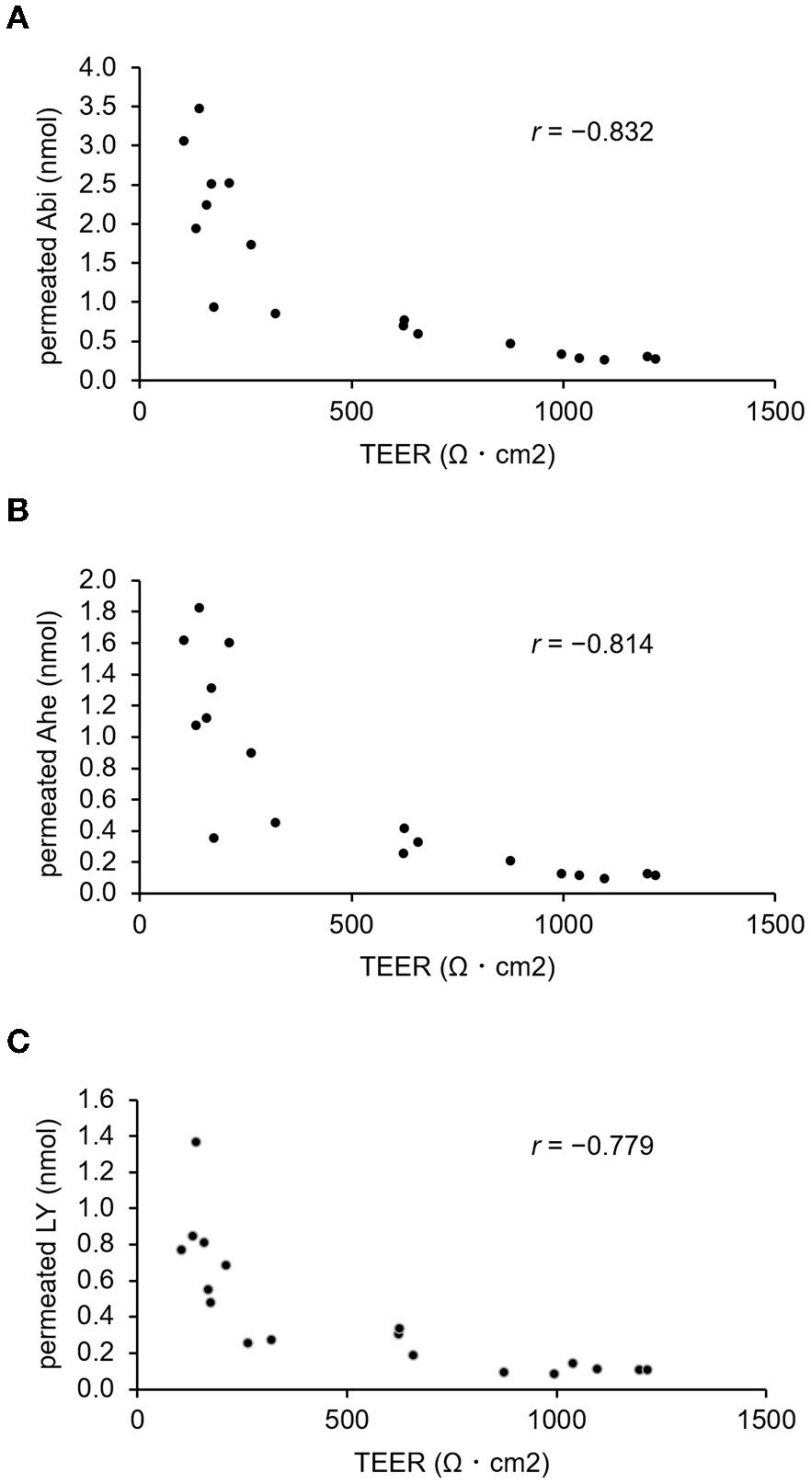
Correlation between the amount of permeated AOSs and the TEER value of Caco-2 cell monolayers. The Pearson correlation coefficient value (*r*) between the TEER and the amount of Abi **(A)** or Ahe **(B)** that permeated the Caco-2 cell monolayers is compared with that of Lucifer yellow **(C)**.

The correlation between TEER and permeation rate was examined to confirm the occurrence of Abi and Ahe transport *via* the paracellular pathway by using the LY fluorescent stain for comparison. Thus, Abi, Ahe, and LY were added to the apical chamber with or without cytochalasin B. The basal chamber solution was collected, and the TEER was measured every 1 h for 3 h, thereby quantifying Abi, Ahe, and LY in the basal chamber. The TEER and quantified values are plotted as a scatter plot in Figure 6. The Pearson correlation coefficient value of Abi or Ahe was comparable to that of LY (Abi, −0.832; Ahe, −0.814; LY, −0.779).

## Discussion

This study demonstrated that orally administered AOSs, which are derived from agar, permeated rat plasma. Further, *in vitro* experiments revealed that the AOSs could cross the Caco-2 cell monolayers *via* the paracellular pathway. The plasma samples were derivatized with ABEE to determine the plasma concentration of the orally administered AOSs, which could detect Abi, Ate, and Ahe sensitively and selectively. After orally administering AOSs to the rats, the Abi, Ate, and Ahe concentrations in the plasma were detected. The amount of detected AOSs increased in a time-dependent manner, with an inverse correlation between the plasma detection levels and the MW of the AOSs. *In vivo* pharmacokinetic studies of oligosaccharides or polysaccharides have been conducted previously. For example, in rats, orally administered chitosan oligosaccharides with lower MWs enhance the detection levels (15), which is consistent with our results.

Generally, orally administered carbohydrates are catalyzed to disaccharides by amylase in the saliva and subsequently to monosaccharides by disaccharidases in the brush border membrane. Known transporters of monosaccharides are the sodium–glucose transporter (SGLT) and facilitative glucose transporter (GLUT) families (16, 17). Agarase, the enzyme which hydrolyzes AOSs, is primarily found in marine bacteria, including *Pseudomonas atlantica* (18), *Vibrio* sp. (19), and *Pseudoalteromonas* spp. (20). However, AOSs do not degrade in the mammalian gastrointestinal tracts due to the absence of agarase. This study showed that a metabolic inhibitor (sodium azide) does not inhibit the permeation of Abi or Ahe through Caco-2 cell monolayers. Therefore, AOSs can permeate Caco-2 cell monolayers independently of SGLTs or GLUTs without being degraded by enzymes. In addition, the transport of AOSs was not inhibited by colchicine, which can inhibit intracellular transport (21). Agaro-oligosaccharides were not detected from the cell lysate after the transport experiment (data not shown), suggesting that the transport of AOSs might not proceed *via* the intracellular pathway. In contrast, the permeation of Abi and Ahe was enhanced by treatment with cytochalasin B, a tight junction inhibitor. Furthermore, the Pearson correlation coefficient value of Abi or Ahe is similar to that of LY, a paracellular marker. These results suggest that Abi and Ahe mainly permeated *via* the paracellular pathway.

Paracellular pathway is one of the four distinct transport routes, where tight junctions form an intercellular diffusion barrier between cells, for restricting the transport of macromolecular substances. On the other hand, the paracellular pathway is the preferred route for the transport of hydrophilic substances, including bioactive peptides (22–24) and human milk oligosaccharides (25). The transport mechanism of the peptides GIGLP, RLSFNP, RVPSL have been investigated using inhibitors of transcytosis and tight junction. The transport rate of these peptides was only modulated after the treatment of tight junction inhibitors. Tight junctions are composed of multiple proteins such as claudin, occludin, zonura occludens-1, and junction adhesion molecule (26). Among tight junction proteins, the structure and function of occludin are most affected by cytochalasin treatment (27), suggesting that permeation of AOSs might be related to decreased occludin. Further investigation using siRNA-mediated knockdown (28) will be needed to elucidate the molecular mechanisms underlying the permeation of AOS.

AOSs exhibit anti-inflammatory activity both *in vitro* and *in vivo*. Enoki et al. (6) demonstrated that oral administration with Abi and Ate to mice resulted in the suppression of nitric oxide (NO) production on stimulated peritoneal macrophages. In this study, mice were treated with 3% AOSs containing water for 3 weeks. Considering the average water consumption of a mouse (30 g mouse, 5 ml water consumption per day), the applied dose meant the intake of 150 mg of AOSs per day, which was consistent with 5.0 g/kg body weight per day of orally administered AOSs. This dosage was same as that used in our study. The AUC and Cmax of AOSs was not significantly detected; however, AOSs might interact with the peritoneal macrophages directly. Future research will be required to assess the absorption rate of orally administered AOSs into human circulation.

The activity of AOSs may depends on the structure of the reducing end. Enoki et al. (5) revealed that neoagarohexaose, which has Gal at the reducing end, exhibited no anti-inflammatory activity, indicating that AnGal at the reducing end is essential for the biological function of AOSs. In addition, there was no relationship between the inhibitory effect on the amount of NO produced and the size of AOSs (including Abi and Ahe) *in vitro*. No information regarding the distribution of AOSs in peripheral tissues is available. Therefore, it could be necessary to determine the distribution of AOSs in different tissues and investigate the degree of bioaccumulation to understand the mechanism underlying the biological effects associated with the molecular size.

## Conclusion

The present study evaluated the oral bioavailability and intestinal permeability of AOSs, including Abi, Ate, and Ahe. We demonstrated that orally administered AOSs were detected as their intact forms in the plasma of rats, which were dose-dependently and negatively correlated with MW. Low-MW Abi showed higher permeation compared with high-MW Ahe across Caco-2 cell monolayers, which was consistent with the *in vivo* results. The inhibitors cytochalasin B increased the transport of AOSs across Caco-2 cell monolayers, which implied that the route of transport of AOSs mainly rely on paracellular pathway. These results suggest that orally administered AOSs can be absorbed *via* the gastrointestinal tract into the circulating blood and might exhibit their biological activities in the peripheral tissues such as joints and skin in an intact form. The current findings highlight the potential of the application of AOSs, especially Abi, in the formulation of functional foods or pharmaceuticals.

## Data availability statement

The original contributions presented in the study are included in the article/Supplementary material, further inquiries can be directed to the corresponding author.

## Ethics statement

The animal study was reviewed and approved by Institutional Animal Care and Use Committee of Shinshu University.

## Author contributions

IS, KK, YS, HM, and SK conceived and designed the study. IS, YK, YI, and HM performed the experiments. IS, YI, and HM analyzed the data. IS and HM wrote the manuscript. YS and SK revised the manuscript. All authors read and approved the final version of the manuscript.

## Conflict of interest

Authors IS and KK are employees of Ina Food Industry Co., Ltd. The remaining authors declare that the research was conducted in the absence of any commercial or financial relationships that could be construed as a potential conflict of interest.

## Publisher's note

All claims expressed in this article are solely those of the authors and do not necessarily represent those of their affiliated organizations, or those of the publisher, the editors and the reviewers. Any product that may be evaluated in this article, or claim that may be made by its manufacturer, is not guaranteed or endorsed by the publisher.

## Abbreviations

ABEE: *p*-aminobenzoic ethyl ester
Abi: agarobiose
Ahe: agarohexaose
AOSs: agaro-oligosaccharides
Ate: agarotetraose
AUC: area under the curve
Cmax: maximum observed plasma concentration
DL: desolvation line
DMSO: dimethyl sulfoxide
E-MEM: Eagle's minimal essential medium
FBS: fetal bovine serum
GC-SPE: graphitized carbon solid-phase extraction
GLUT: glucose transporter
HBSS: Hank's balanced salt solution
HMOs: human milk oligosaccharides
LMW-CS: low-molecular-weight chondroitin sulfate
LNnt: lacto-*N*-neotetraose
MEM-NEAA: MEM non-essential amino acids
LY: Lucifer yellow
MTT, 3-(4, 5-dimethylthiazol-2-yl)-2: 5-diphenyltetrazolium bromide
NO: nitric oxide
PS: penicillin–streptomycin solution
SGLT: sodium–glucose transporter
TEER: transepithelial electrical resistance
Tmax: time at maximal concentration.
