# Supplementary material for: Intestinal permeability of agaro-oligosaccharides: Transport across Caco-2 cell monolayers and pharmacokinetics in rats
Source: Front Nutr. 2022 Sep 16;9:996607. doi: 10.3389/fnut.2022.996607 (PMC9525106; doi:10.3389/fnut.2022.996607)
Supplement: Supplementary file 1 [file Data_Sheet_1.PDF]

Supplementary Material

Supplementary Table 1. ^{13}C-NMR data of agarobiose (Abi) and agarotetraose (Ate) in D_2O.

Residue	Carbon number	^{13}C chemical shift	
		Abi	Ate
External D-Galactose (Gal) residue	1	102.37	102.77
	2	70.58	71.09
	3	72.50	73.23
	4	68.52	69.21
	5	75.25	75.99
	6	61.05	61.66
Internal 3,6-anhydro-L - Galactose (AnGal) residue	1		98.62
	2		69.89
	3		80.21
	4		77.46
	5		75.58
	6		69.54
Internal Gal residue	1		102.82
	2		70.50
	3		82.42
	4		68.88
	5		75.51
	6		61.66
External AnGal residue	1	89.76	90.47
	2	72.50	73.23
	3	82.77	83.46
	4	85.53	86.28
	5	75.09	75.81
	6	72.94	73.66

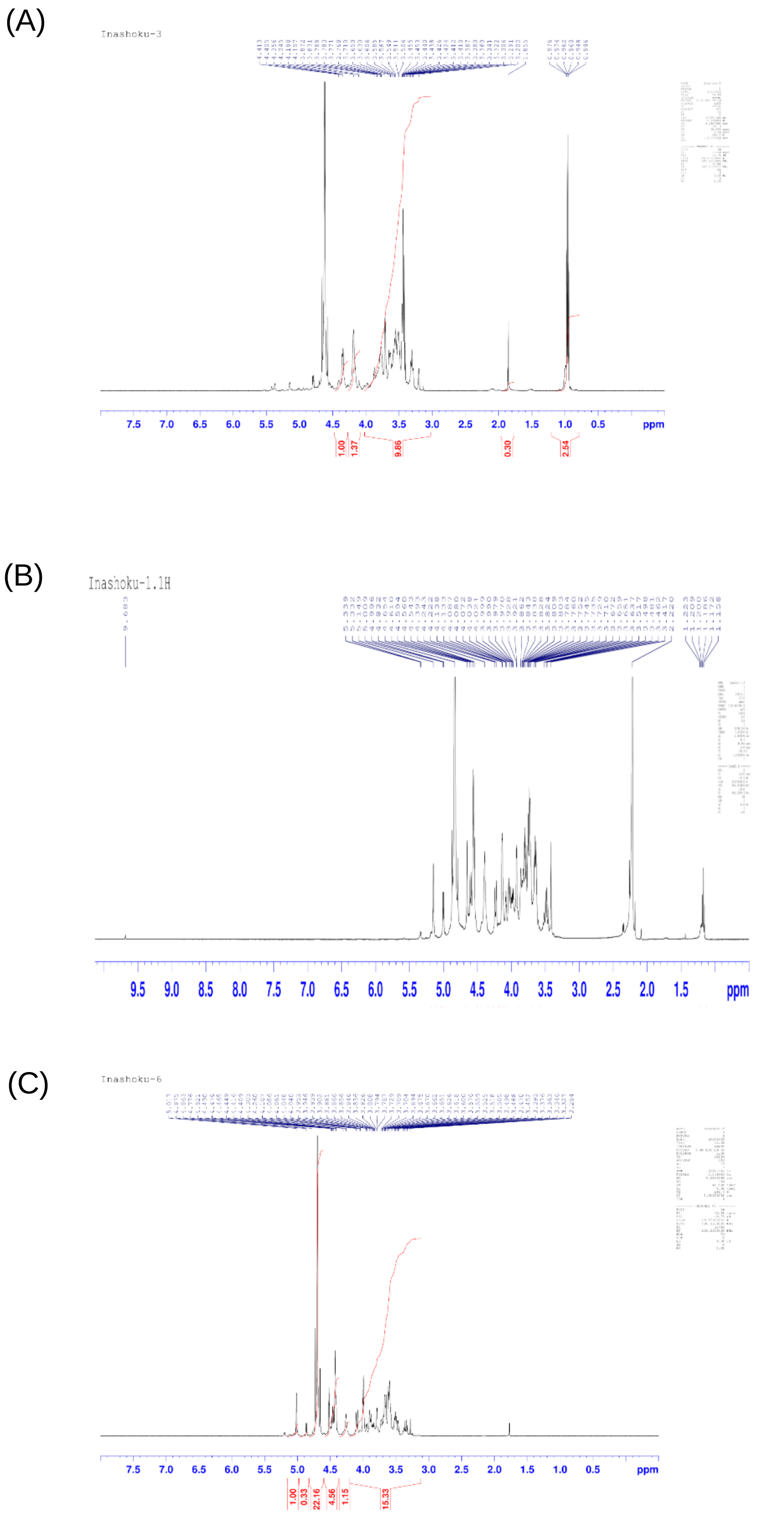

Supplementary Figure 1. ^{1}H-NMR spectra of Abi **(A)**, Ate **(B)**, and Agarohexaose (Ahe) **(C)**.

(A)

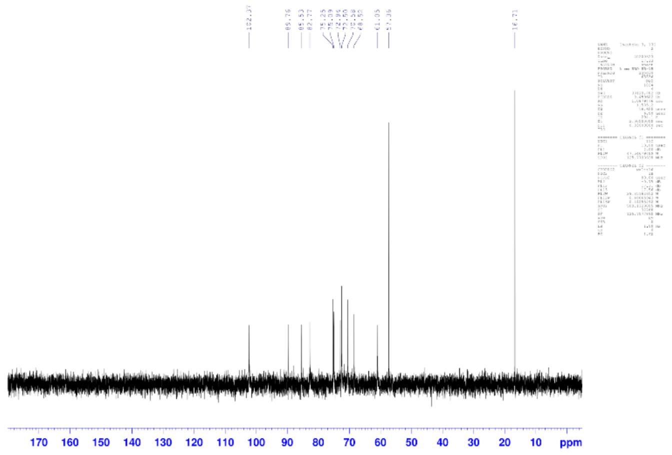

(B)

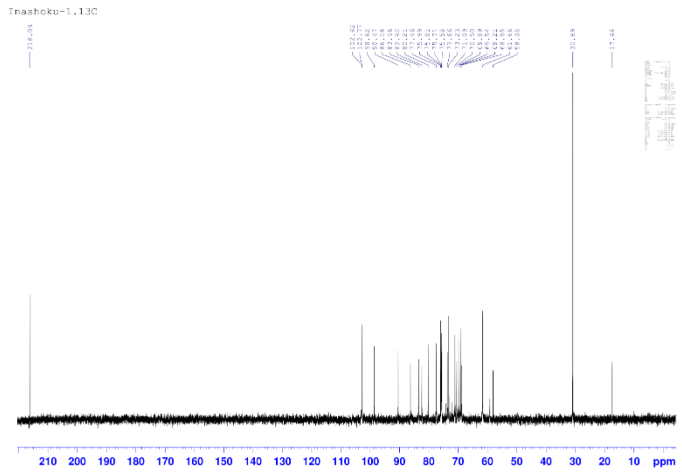

(C)

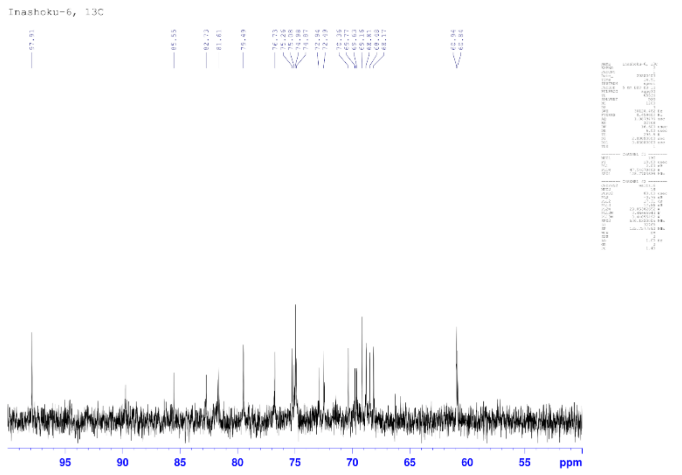

Supplementary Figure 2. ^{13}C-NMR spectra of Abi **(A)**, Ate **(B)**, and Ahe **(C)**.

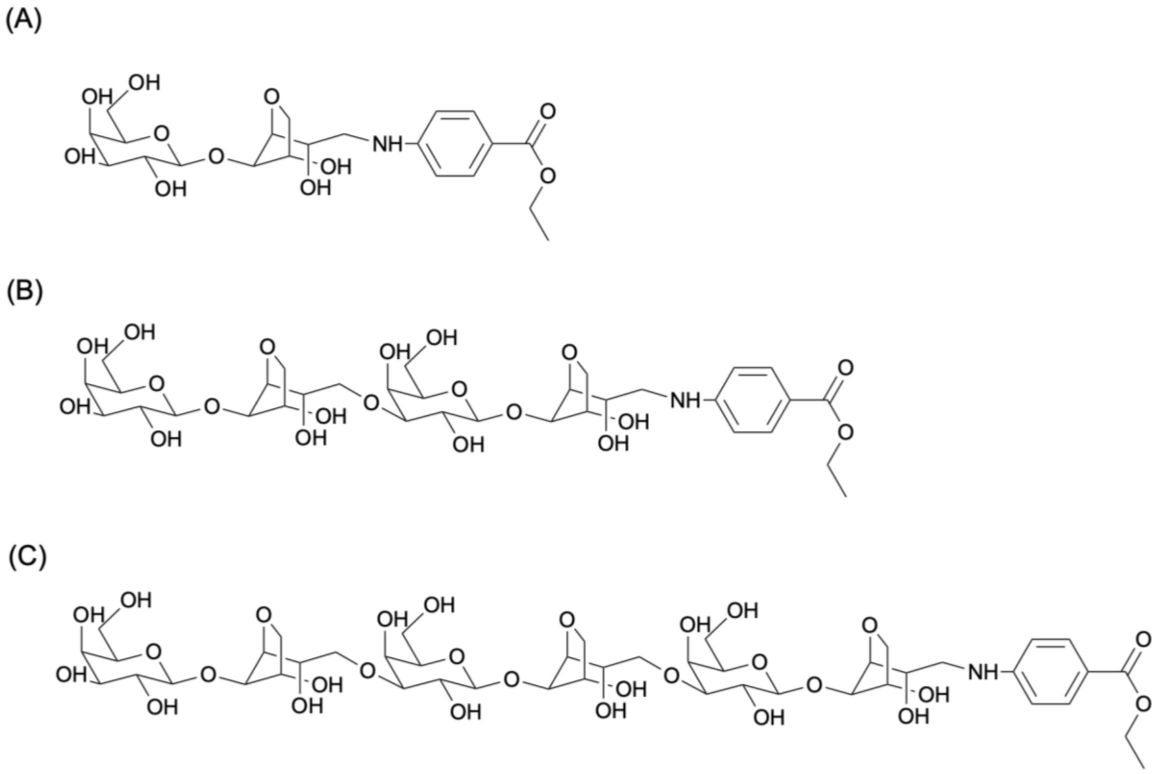

Supplementary Figure 3. Structures of *p*-aminobenzoic ethyl ester-derivatized agaro-oligosaccharides (ABEE-AOSs). AOSs were converted to ABEE at their reducing ends, producing ABEE-Abi **(A)**, ABEE-Ate **(B)**, and ABEE-Ahe **(C)**.

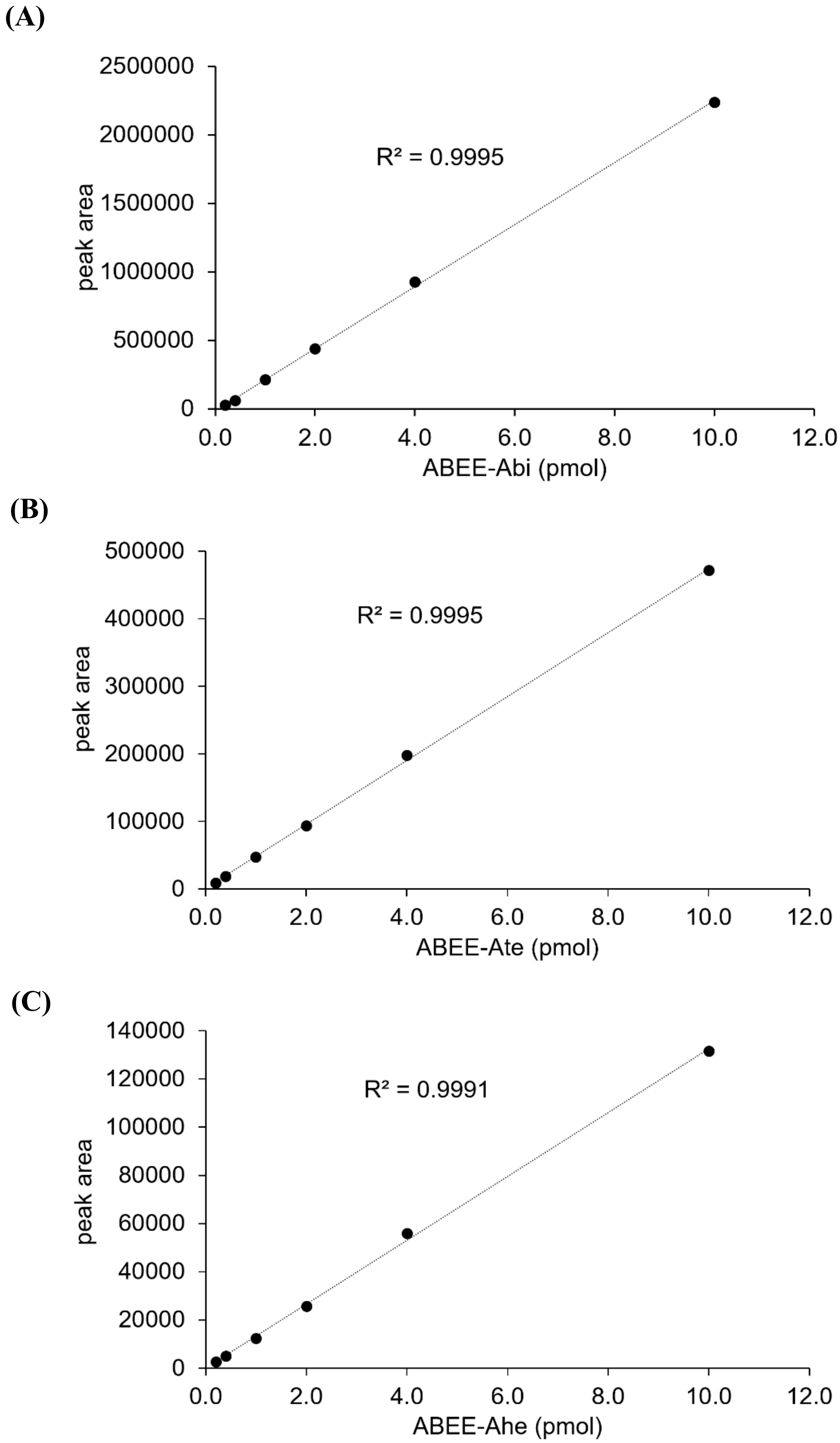

Supplementary Figure 4. Standard curves of ABEE-Abi (A), ABEE-Ate (B), and ABEE-Ahe (C) obtained by high-performance liquid chromatography–electrospray ionization mass spectrometry.
